# What Victims of Domestic Violence Experience in Emergency Departments; a Cross Sectional Study

**Published:** 2019-11-24

**Authors:** Brieana Rodriguez, Madalyn Mandich

**Affiliations:** 1 Medical College of Wisconsin, Milwaukee, Wisconsin, USA.

**Keywords:** Domestic violence, emergency service, hospital

## Abstract

**Introduction:**

Domestic violence (DV) is a problem defined as physical, sexual, and/or mental abuse used by one person in a relationship in order to gain control over the other. This study aimed to investigate what victims of domestic violence are experiencing when they present to the emergency department (ED).

**Methods:**

The survey was conducted during the women's initial visit to Sojourner Family Peace Center (SFPC) in Milwaukee, WI. It included 22 questions assessing women’s encounters with screening and treatment for domestic violence in the ED.

**Results:**

24 surveys were collected over 7 months. Thirteen women presented for treatment of injuries related or not related to abuse. Problems with abuse-related care they received were identified. 31% of women presenting with obvious signs of abuse, such as human bite wounds or head injury, were not screened. Four of 11 women were screened with family or law enforcement present. Nine of 11 were screened by a nurse, social worker, or police officer, not a physician. Four women felt rushed by healthcare professionals and that they did not genuinely care. Most noticeably, women were not screened at all.

**Conclusion:**

DV screening must be done with no family present. Removing law enforcement from rooms is noted to make women feel more comfortable. If a family member is adamant about remaining with the patient, the patient can be removed for a blood draw or sensitive exam to attain privacy. Standardizing screening could aid in making DV victims feel less rushed and more at ease. The courageous women sharing personal stories help pave the way to better treatment for future victims of DV presenting to the ED.

## Introduction:

Domestic Violence is a continuing and devastating problem defined as physical, sexual, and/or mental abuse used by one person in a relationship in order to gain control over the other. Over 10 million people in the United States will experience domestic violence at some point in their lifetime, and an estimated 34% will seek medical care for their injuries (1). Domestic violence victims seeking care will likely present to primary care clinics, urgent care clinics, OB/GYN clinics, EDs, and almost every other specialty for treatment and help. 

Domestic violence is an issue all healthcare professionals will encounter at some point throughout their career. It is imperative that healthcare professionals are trained to adequately and respectfully identify and treat patients presenting to them for domestic violence related health problems. This is an issue so vital that the Joint Commission (2), the American College of Emergency Physicians (3), and the US Preventive Services Task Force (4) have strongly encouraged screening for domestic violence. However, even after years of medical organization’s advocacy, only about 30% of women who presented to the Emergency Department have actually been screened (5). 

This is in contrast to physicians caring for pregnant women who report routine screening 11-39% of the time, no matter the healthcare setting (6). Numerous factors play a role in this process and all need to be taken into consideration when screening for this sensitive topic. For example, it must be taken into account where the conversation takes place, how it takes place, and the appropriate response or follow up to anything a domestic violence victim discloses. Studies have shown that this process, which is supposed to be meaningful and private, is often hindered by having a third party present, lack of referral and appropriate resources, and lack of empathy (7). In order to gain a better understanding of this process and the pitfalls that are continuing to occur, it is timely and of critical importance to get the perspective of the women who are in this vulnerable position. 

Previous studies involving targeted interventions toward pregnant and postpartum women found decreased episodes of domestic violence, reduced reproductive coercion, and improved birth outcomes (6). This evidence of a positive effect of domestic violence screening in an obstetrics setting warrants further investigation in the ED setting as they are often the healthcare setting that victims initially present to for care, safety, and treatment. In addition, the unique characteristics of the ED setting call for both helping victims, as well as, taking extra caution to provide such care due to the acute nature of the visit. Due to this, focusing on the ED will provide valuable information that can set a basis for all other healthcare settings when screening for domestic violence. For the above-mentioned reasons, this study aimed to ivestigate what victims of domestic violence are experiencing when they present to the ED. 

## Methods:

***Study design and setting***

This cross sectional study was conducted from November 2018 to June 2019 on the clients of Sojourner Family Peace Center in Milwaukee, WI, who reported presenting to the ED for abuse-related or non-abuse-related services. Sojourner Family Peace Center is the largest nonprofit provider located in Wisconsin, serving over 9,500 victims each year (8). They offer resources, such as helping obtain a restraining order and providing a case manager, that help women affected by domestic violence receive justice. In addition to legal help, they also offer an ample amount of references and follow-up care to aid women in gaining independence and self-sufficiency once they leave the safety and shelter of Sojourner Family Peace Center. Due to the significant amount of care Sojourner Family Peace Center provides to the women of Southeastern Wisconsin, we decided to partner with them in order to gain first-hand insight into what victims of domestic violence are experiencing when they present to the ED through a 22 questions survay.

In order to keep the data secured and to protect confidentiality, no personal health information was stated on the survey. The information gathered was anonymous and any woman who had not previously presented to the Emergency Department was excluded. The methods of study were reviewed and approved by the Institutional Review Board, and the data from this study will be used to develop recommendations for improving ED treatment of domestic violence victims. 

***Participants***

All females aged 18 years and older who presented to Sojourner Family Peace Center for an initial interview process, regardless of whether or not they had ever presented to an Emergency Department for treatment of injuries sustained due to domestic violence, were included. This is in congruence with the guidelines that Sojourner Family Peace Center has for individuals who qualify for their interview process.

***Data gathering***

The survey included 22 questions, ranging from yes/no to open-ended (Appendix 1). The questions directly measured the opinion of the women and their encounters with the screening process for domestic violence, as well as the treatment for their symptoms in any Emergency Department visit. The women also provided their opinion on any improvements they believed could be made during their entire visit to an Emergency Department. 

The survey was conducted during the women's initial visit to the center. The written survey was completed by the participant with the help (if needed) of a Sojourner representative during their in-person interview process. Our survey was placed within the interview Sojourner conducts for their own purposes. It was presented to the woman interviewed as being part of one long question process. 

Data gathering and interviews were done by Dr. Erin Schubert, Director of Outcomes & Evaluation at Sojourner Family Peace Center. Dr. Erin Schubert is also currently the Sojourner Representative who completes each interview Sojourner distributes. Dr. Schubert is trained in performing interviews, distributing surveys, and working with abuse victims. Dr. Erin Schubert made it clear to the participants that, this survey was a voluntary research activity distinct from the rest of the standard interview. If the domestic violence survivor reported having been to an Emergency Department for abuse-related or non-abuse-related care since her last interview/survey completion, we conducted another survey. If she had not, then no survey was conducted. Each of these interviews were independent of each other and any previous visit to the Emergency Department did not play a role in a subsequent visit. This follow up interview/survey was again anonymous. 

The interviews were conducted and recorded by Erin Schubert in a private room at Sojourner. The surveys were then stored in a locked, limited access area, available to only Dr. Erin Schubert and minimal Sojourner Representatives. The surveys were stored separately from any other forms or surveys Sojourner distributes. 

## Results:

24 surveys were collected over the course of 7 months. Thirteen women presented to the ED for treatment of physical injuries related or not related to abuse. The remaining 11 denied ever presenting to an ED for treatment related to injuries from abuse. Ten out of the thirteen (77%) women who completed surveys identified problems with the abuse-related care they received. For example, women were screened for domestic violence while a family member was present in the room with them. Others were screened while law enforcement was present. Most were screened by either a nurse, social worker, or police officer, not a physician. A handful of women felt as though they were being rushed by their healthcare professionals and that they did not genuinely care. However, the most noticeable complaint was that these women were not screened at all. 

Eleven women were screened for domestic violence when they presented to the Emergency Department. Of these eleven, seven were screened privately, three were screened with another family member present in the room with them, and 1 was screened with her husband, the abuser, in the room. 

Amber recalled an encounter while her husband was in the room. The doctor just “asked me about my knee. I said I fell down.” Even if she had been able to speak about it, “I was totally unaware that anyone could have even helped me. I would have needed someone to clue me in.” 

Three out of the 13 women who presented to the Emergency Department stated they were not comfortable being around, or talking to people in law enforcement. They stated that they had negative attitudes about police, and that they felt that the police did not care about them or their safety. 

Comments from Brianna stated the following. “Police = bad. Didn’t care. Didn’t believe me. Took forever to get to the house then they didn’t even arrest him.” “It wasn’t their fault (doctors/nurses) at all but keep that officer outta there.”

One woman in particular, Chelsea, stated the following in regards to law enforcement:

“I went there a few times. The nurse woman who was asking all the questions about my relationships so I could tell that they were suspicious and kind of know what was up. She just turned around her badge and was telling me how she dates a guy in the police and all that. She was very nice and open and all that. I just wasn’t going to open up to them. My ex-fiancé was so smart and manipulative and was in law enforcement so who knows who he knew and what they were telling him”

Four women reported they either felt rushed while being screened or that the person screening them did not genuinely care. One also felt as though the provider screening them felt uncomfortable asking the questions. 

Diana reported, “I did feel comfortable at first and told them all that until I realized they really didn’t care and weren’t believing so then I was just like okay whatever.”

Another comment from Eva stated, “I would’ve felt safer if they took their time and didn’t rush. I mean I understand they got other patients and all that, but it just takes a minute to ask.”

Francesca recalls, “They did ask a lot of questions. It seemed like they were sometimes trying to avoid the subject or avoid crossing the line or something.” 

During an encounter Giovanna remembers, “They just sent me on my way like they don’t care at all about it.”

Out of the 13 women who presented to the ED with symptoms related to domestic violence; four were not screened. 

This included Heidi who reported the following: “I was pistol whipped and had a split open head. No one there asked me about DV.”

As well as Ingrid who stated, “I had a sprained ankle and sprained wrist from a domestic violence incident. When I went in, I just said I fell down the stairs doing laundry.”

Jennifer also recalled, “My husband and I were fighting and he bit my finger, so I went in. Just asking me instead of brushing it off. I mean I had a human bite on me…like DUH!”

Two women stated they were not given any community resources to contact following their stay in the Emergency Department and they felt as though if they would have had received resources it would have been extraordinarily helpful and maybe they would not have had to go back to an abusive home after discharge. 

And finally, Kalie stated the following: “Maybe if they could provide some sort of housing information or funds so I didn’t feel like I had to go back to home.’

## Discussion:

From this data collection, we have started to gain valuable insight on the interactions between healthcare personnel and victims of domestic violence in the Emergency Department. The most valuable information is that there is still the possibility of women presenting with obvious symptoms of abuse, such as a human bite wound or head injury, not being screened. Presenting to an Emergency Department with a head injury or human bite wound should warrant screening of domestic violence. Another important piece of information we have gathered is that when screening is done, it is often not completed in a private or secure manner. The majority of screening is being done by either a nurse, social worker, or law enforcement. It is rarely being done by physicians. While the sample size gathered may be small, the 13 survivors surveyed represent a significant portion of women who have experienced domestic violence and have beneficial insight to help improve care and treatment in the Emergency Department. Even with the relatively small sample size, it is evident that screening for domestic violence is dire and needs to occur more often than what is currently happening. Our research shows the importance of continuing research on domestic violence screening and what can be done to improve care in the Emergency Department. These courageous women who have shared their personal stories with us can help pave the way to better treatment for future victims of domestic violence who present to the Emergency Department hoping for care and safety. From this collaboration, we hope to gain information that will describe the ways Emergency Department personnel interact with domestic violence victims when providing care. This information will help us identify desirable behaviors of physicians and other professional care givers, as well as ways they can improve their behavior/attitude/treatment of domestic violence victims when they present to the Emergency Department. This data will help us understand what is working well and what needs improvement in regards to domestic violence victims in the Emergency Department. We hypothesize that there will be characteristics of the Emergency Department that are working well, but that there will also be areas that need improvement to provide the most beneficial care for domestic violent victims during their visit. 

We recommend universal screening of domestic violence to all women who present to Emergency Department. This screening must be done privately. It is extremely important to screen patients secluded from people who may influence their conversations and safety. There are multiple opportunities for healthcare professionals to pull their patients in a private room in order to make them feel safe enough to open up. If a family member or abuser is adamant about not leaving the patient’s side, then personnel can tell them they need the patient for an x-ray, blood draw, or sensitive exam in order to get the patient alone for an opportunity to screen them. Domestic violence screening should always be done in a safe environment, private room, with no other family members or personnel with them. Most victims of domestic violence do not feel comfortable interacting with law enforcement and removing them from the patient’s room could be the first step to making the women feel safe in the Emergency Department and possibly allow them to open up about their experiences. Standardizing who screens patients for domestic violence may be a way to properly train healthcare personnel to adequately and respectfully screen. This could also help improve the way personnel ask the questions to hopefully make the domestic violence victim feel less rushed and more at ease. Having an appointed person or advocate to screen for domestic violence will also ensure that every person who presents to the Emergency Department is screened. This advocate may then be able to stay with a woman who has confirmed she is a victim of domestic violence so she can feel safe throughout her entire stay in the Emergency Department. They can also present standardized community resources to ensure her safety following her discharge from the Emergency Department.

***Limitations***

Limitations to this study include the small sample size. There was a relatively lower number of women who presented to Sojourner Family Peace Center for initial interviews during the time period of our data collection.

## Conclusion:

We gained valuable insight on interactions between healthcare personnel and DV victims. DV screening must be done with no family present. Removing law enforcement from rooms is recommended to make women feel more comfortable. If a family member is adamant about remaining with the patient, the patient can be removed for a blood draw or sensitive exam to attain privacy. Standardizing screening could aid in making DV victims feel less rushed and more at ease. The courageous women sharing personal stories help pave the way to better treatment for future victims of DV presenting to the ED.
